# *Streptococcus pneumoniae* binds collagens and C1q via the SSURE repeats of the PfbB adhesin

**DOI:** 10.1111/mmi.14920

**Published:** 2022-05-30

**Authors:** Giuseppe Valerio De Gaetano, Francesco Coppolino, Germana Lentini, Agata Famà, Chiara Cullotta, Ivana Raffaele, Chiara Motta, Giuseppe Teti, Pietro Speziale, Giampiero Pietrocola, Concetta Beninati

**Affiliations:** ^1^ Department of Human Pathology University of Messina Messina Italy; ^2^ Department of Biomedical, Dental and Imaging Sciences University of Messina Messina Italy; ^3^ Department of Molecular Medicine University of Pavia Pavia Italy; ^4^ Charybdis Vaccines Srl Messina Italy; ^5^ Scylla Biotech Srl Messina Italy

## Abstract

The binding of *Streptococcus pneumoniae* to collagen is likely an important step in the pathogenesis of pneumococcal infections, but little is known of the underlying molecular mechanisms. Streptococcal surface repeats (SSURE) are highly conserved protein domains present in cell wall adhesins from different *Streptococcus* species. We find here that SSURE repeats of the pneumococcal adhesin plasminogen and fibronectin binding protein B (PfbB) bind to various types of collagen. Moreover, deletion of the *pfbB* gene resulted in a significant impairment of the ability of encapsulated or unencapsulated pneumococci to bind collagen. Notably, a PfbB SSURE domain is also bound to the complement component C1q that bears a collagen‐like domain and promotes adherence of pneumococci to host cells by acting as a bridge between bacteria and epithelial cells. Accordingly, deletion of PfbB or pre‐treatment with anti‐SSURE antibodies markedly decreased pneumococcal binding to C1q as well as C1q‐dependent adherence to epithelial and endothelial cells. Further data indicated that C1q promotes pneumococcal adherence by binding to integrin α_2_β_1_. In conclusion, our results indicate that the SSURE domains of the PfbB protein promote interactions of pneumococci with various types of collagen and with C1q. These repeats may be useful targets in strategies to control *S. pneumoniae* infections.

## Graphical abstract

We show here that the SSURE domains of the bacterial cell wall adhesin PfbB contribute to the ability of pneumococci to bind various types of collagens and C1q, a complement component. We also show that C1q acts as a bridge between PfbB SSURE domains and the α_2_β_1_ integrin thereby promoting adherence to, and invasion of, endothelial and epithelial cells. Targeting SSURE domains may be advantageous in alternative strategies to control pneumococcal infections.

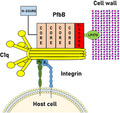

## 1 INTRODUCTION

*Streptococcus pneumoniae* (or the pneumococcus) is an encapsulated Gram‐positive organism that is the main cause of community‐acquired pneumonia and other frequent infections, such as otitis media and meningitis (Bogaert et al., 2004). Although pneumococci asymptomatically colonize the upper airways, they frequently shift from a commensal to a pathogenic condition within the host. It has been estimated that almost 1 million children below 5 years of age die from, and 15 million become ill with, pneumococcal disease (O'Brien et al., 2009). The burden of this disease is also high in the elderly and patients with associated immunodeficiencies (Picard et al., 2003). Bacteria make use of various strategies to promote their adhesion to eukaryotic cells during colonization and pathogenesis. Most Gram‐positive pathogens express cell‐wall surface structures, which directly or indirectly enhance bacterial interactions with host tissues, including cell adherence and invasion (Kline et al., 2009). Extracellular matrix (ECM) components, such as collagens, fibronectin, vitronectin, fibrinogen, and plasminogen, represent fundamental targets for interactions of bacteria with host cells (Bingham et al., 2008; Binsker et al., 2017; Hammerschmidt et al., 2019; Singh et al., 2012).

Collagens, the most abundant components of ECM and the main constituents of connective tissues, are also frequently targeted by pathogenic microbes. Collagens consist of the association of three polypeptide α chains that give rise to several molecular isoforms. The 28 types of collagen identified so far include fibril‐forming and network‐forming collagens. Fibril‐forming collagens, such as type I, II, and III, are assembled into triple‐helical strands during connective tissue formation and wound healing. Network‐forming collagens, such as type IV, VI, and X, determine the structure of basement membranes and are in direct contact with epithelial, endothelial, and muscle cells (Bella & Hulmes, 2017).

C1q, a component of the complement system, is an important part of innate immune defenses against invading pathogens (Ricklin et al., 2010). The C1q molecule has the shape of a flower bouquet and is formed by six identical trimeric proteins that associate to form the “stalk” and then diverge to form six “stems” each ending in a globular “flower head” (Agarwal & Blom, 2015). Each of the six proteins consists of three polypeptides whose collagen‐like domains form a characteristic triple helix structure. The globular flower head has the function to bind antibodies as well as some acute‐phase proteins, while the stalk binds to the C1r and C1s components of the complement system. The binding of the globular region to pathogen‐bound antibodies or acute phase proteins induces a conformational change of C1r and activates the classical complement activation pathway (Agarwal & Blom, 2015). At least two surface proteins with C1q‐binding properties have been identified in *S. pneumoniae*, namely the peptidoglycan‐associated glycolytic enzyme glyceraldehyde‐3‐phosphate dehydrogenase (GAPDH) (Terrasse et al., 2012; Terrasse et al., 2015) and pneumococcal endopeptidase O. Binding of the latter to C1q can promote bacterial adherence to and invasion of epithelial cells (Agarwal et al., 2013) and modulate activation (Agarwal et al., 2014).

Collagen‐binding proteins of several species of staphylococci and streptococci have been studied in detail (Arora et al., 2021; Philominathan et al., 2012). Less is known about the ability of *S. pneumoniae* to bind collagen and of the functional significance of this interaction. This pathogen was found in an early study to bind collagen type I and IV in a strain‐dependent manner (Kostrzynska & Wadstrom, 1992). Moreover, collagen VI has been proposed as a putative adhesive target for pneumococcal binding to respiratory epithelium, even though the capacity of surface pneumococcal adhesins to bind this type of collagen has been incompletely characterized (Bober et al., 2010). A few proteins with type I collagen‐binding activity have been described in *S. pneumoniae*. The tip protein of the pneumococcal pilus‐1, RrgA, has been demonstrated to bind to collagen I as well as laminin (Hilleringmann et al., 2008). Using atomic force microscopy, the pilus backbone protein RrgB was also found to bind to human collagen I in a manner influenced by the orientation of collagen fibrils (Becke et al., 2019). Moreover, DiiA (Dimorphic invasion‐involved A), a novel pneumococcal dimorphic cell wall protein involved in invasive disease, is capable of binding collagen I (Escolano‐Martinez et al., 2016). However, the precise contribution of these proteins to the overall ability of pneumococci to bind collagen has not been assessed.

**FIGURE 1:**
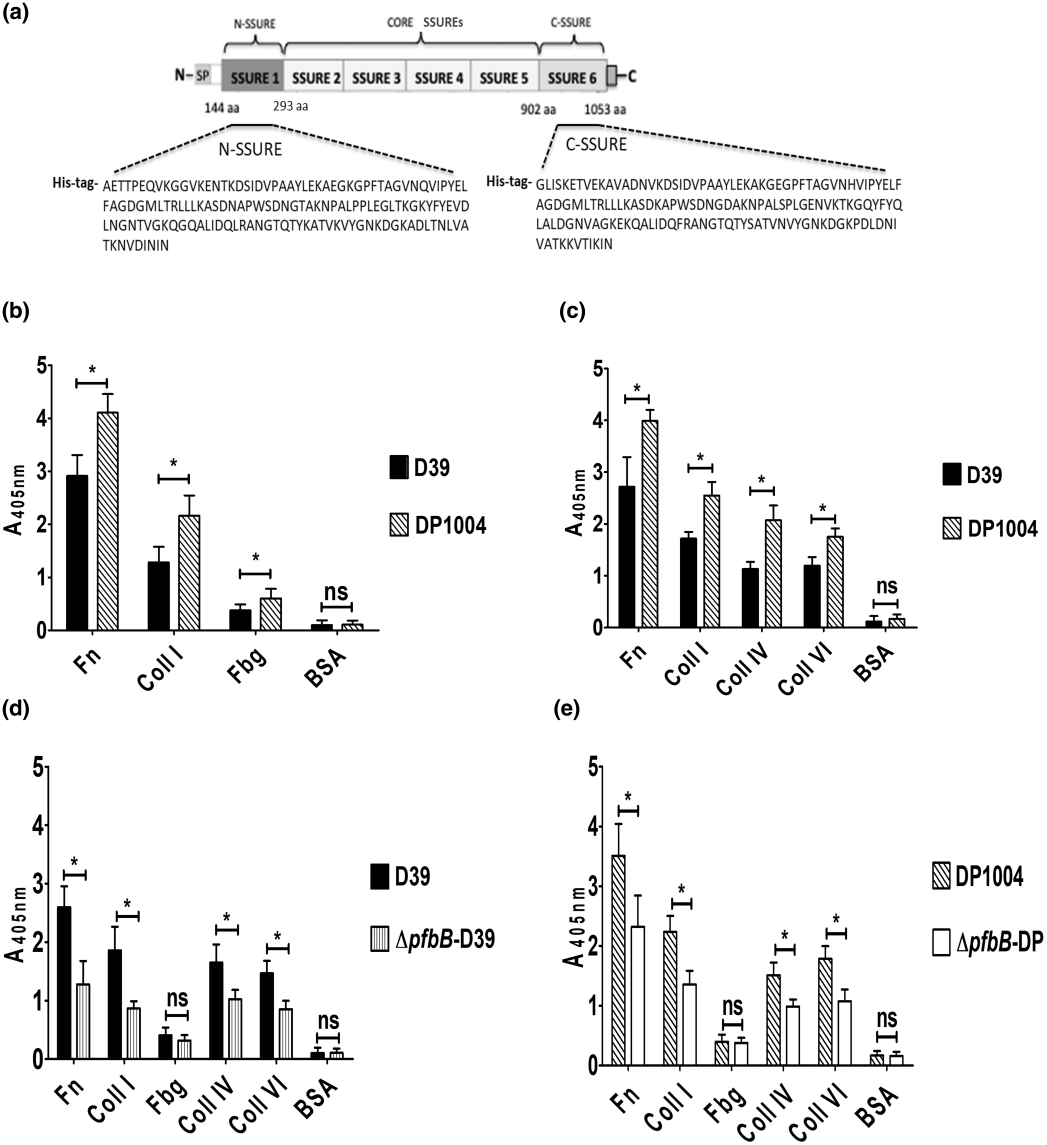
*S. pneumoniae adhesion* to collagens. (a) Schematic representation of the primary structure of PfbB, displaying a region (amino acid residues 144–1053) containing six SSURE repeats and including N‐SSURE (N‐terminal repeat or SSURE 1), CORE SSUREs (SSURE 2–5), and C‐SSURE (C‐terminal SSURE repeat or SSURE 6). N, N‐terminus; SP, signal peptide; C, C‐terminus; aa, amino acid. Shown are the amino acid sequences fused to a polyhistidine tail (his‐tag) of the recombinant C‐terminal SSURE (C‐SSURE) and N‐terminal SSURE (N‐SSURE) domains used in this study. (b) Adhesion of encapsulated D39 and unencapsulated DP1004 strains to wells sensitized with fibronectin (Fn), fibrinogen (Fbg), type I collagen (Coll I), or bovine serum albumin (BSA), used as control. Bacterial adhesion was detected by ELISA using an anti‐pneumococcus serum. (c) Adhesion of the same strains shown in (b) to collagen type I (Coll I), collagen type IV (Coll IV), collagen type VI (Coll VI), fibronectin (Fn), and bovine serum albumin (BSA), used as control. Bacterial adhesion was detected by ELISA using an anti‐pneumococcus serum. Encapsulated D39 (d) and unencapsulated DP1004 (e) strains were compared to their respective isogenic *pfbB* deletion mutants (Δ*pfbB‐*D39 and Δ*pfbB‐*DP) for their ability to adhere to immobilized collagen type I (Coll I), collagen type IV (Coll IV), collagen type VI (Coll VI), fibronectin (Fn) and bovine serum albumin (BSA), used as control. Bacterial adhesion was detected by ELISA using an anti‐pneumococcus serum. Shown are means ± SDs of three independent experiments conducted in triplicate. *p < 0.05 by Mann–Whitney test.

Streptococcal surface repeats (SSURE) have been described as 148–152 amino acid long tandem repeats in several species of streptococci (Bumbaca et al., 2004). *Streptococcus agalactiae* (group B streptococcus or GBS) expresses on its surface PbsP (standing for plasminogen‐binding surface protein), a cell‐wall protein bearing two SSURE domains (Buscetta et al., 2016). *S. pneumoniae* also expresses a protein named PfbB (plasminogen and fibronectin‐binding protein B) (Papasergi et al., 2010) or PavB (pneumococcal adherence and virulence factor B) (Jensch et al., 2010) containing 2 to 6 tandem SSURE domains (Figure 1a). This protein was previously identified as a significant target of antibody responses in patients with pneumococcal pneumonia and invasive infections (Beghetto et al., 2006). The C‐terminal and core SSURE repeats of PfbB are >95% identical but differ from the N‐terminal repeat (Figure 1a). SSURE‐containing proteins endow streptococci with the ability to bind multiple ligands in the extracellular matrix. Group B streptococcal PbsP can bind to plasminogen and vitronectin (Buscetta et al., 2016; De Gaetano et al., 2018; Lentini et al., 2018) while PfbB can interact with plasminogen, fibronectin and thrombospondin via SSURE repeats (Binsker et al., 2015; Jensch et al., 2010; Kanwal et al., 2017; Papasergi et al., 2010).

The present study was undertaken to better understand the molecular mechanisms underlying the interactions between pneumococci and collagen by focusing on the role of the SSURE‐containing protein PfbB. We found here that the SSURE domains of PfbB can interact with various types of collagen and with C1q. Moreover, the binding of PfbB to C1q allows *S. pneumoniae* to adhere to and invade epithelial and endothelial cells by a mechanism involving the collagen‐binding integrin α_2_β_1_.

## 2 RESULTS

### 2.1 Optimal binding of *S. pneumoniae* to collagen requires PfbB

In initial experiments, we assessed the ability of the reference D39 pneumococcal strain to bind collagen I in comparison with fibronectin (Fn) and fibrinogen (Fbg), two ECM components that were previously shown to interact with these bacteria (Holmes et al., 2001; Speziale et al., 2019; Vassal‐Stermann et al., 2014). In this assay, plates were sensitized with host proteins, incubated with pneumococcal cells, and bacterial adhesion was detected using mouse anti‐pneumococcal serum. *S. pneumoniae*, strain D39, interacted with type I collagen with an adhesion efficiency that was intermediate between Fn and Fbg binding (Figure 1b). In addition, we found that the DP1004 strain, an unencapsulated D39 mutant, adheres to collagen I, Fn, and Fbg with moderately greater efficiency than the parental strain (Figure 1b), indicating that the presence of a capsule partially masks the interaction of bacteria with such ligands. We also tested encapsulated and unencapsulated bacteria for adhesion to other collagen types, such as type VI (Bober et al., 2010), and type IV (Bella & Hulmes, 2017). Pneumococci bound efficiently to the different collagen types and the encapsulated strain showed a significantly reduced adhesion also to these ligands (Figure 1c). We next investigated the role of PfbB, a Fn‐binding adhesin (Papasergi et al., 2010), by analyzing the effects of *pfbB* deletion on pneumococcal binding to collagen. A *ΔpfbB* mutant (Papasergi et al., 2010) was significantly impaired compared to the wild‐type D39 parental strain in its ability to bind to various collagen types and Fn, used as a positive control, but not to Fbg (Figure 1d). Since surface expression of PfbB is partially masked in the encapsulated strain (Papasergi et al., 2010), we also determined the impact of *pfbB* deletion in the unencapsulated strain. As expected, *pfbB* deletion in the background of the unencapsulated DP1004 strain also resulted in a significant reduction of collagen adhering ability (Figure 1e). Overall, these data indicate that the PfbB protein significantly contributes to the ability of pneumococci to bind to collagens.

### 2.2 Role of PfbB in pneumococcal growth on collagen

**FIGURE 2:**
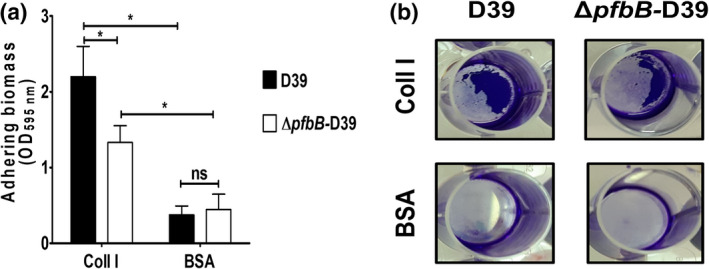
*S. pneumoniae* grows in the sessile form on surfaces coated with type I collagen by a mechanism involving PfbB. (a) Effect of *pfbB* deletion on sessile growth of the encapsulated D39 strain (D39) in wells coated with type I collagen (Coll I) or bovine serum albumin (BSA). Δ*pfbB*, D39 pfbB deletion mutant. Columns indicate optical density at 595 nm (OD_595nm_) of solubilized crystal violet. Shown are means ± SDs of three independent experiments conducted in triplicate. *p < 0.05 by the Mann–Whitney test. (b) Adhering aggregates (purple) of the encapsulated D39 strain and its isogenic *pfbB* deletion mutant (Δ*pfbB*‐D39) before crystal violet solubilization.

Pneumococci can form biofilms in the upper respiratory tract of patients with recurrent infections, a process that is thought to be important in the pathogenesis of the pneumococcal disease (Domenech et al., 2012). Therefore, we investigated whether pneumococci could grow as biofilm‐like aggregates on collagen‐coated surfaces and whether the PfbB adhesin can play a role in the process. To this end, we measured surface‐adherent growth in an assay in which bacteria were resuspended in a fresh culture medium and added to microtiter plates pre‐coated with collagen type I. Pneumococci were then allowed to grow overnight at 37°C. Under these conditions, coating with collagen significantly promoted the growth of wild type D39 bacteria as surface‐adherent aggregates, compared to BSA‐coated control wells (Figures 2a,b). Moreover, bacteria lacking *pfbB* (*ΔpfbB‐*D39) showed a 40% decrease in their ability to form such aggregates on collagen‐coated surfaces relative to the parental D39 strain (Figure 2a). Similarly, *pfbB* deletion significantly decreased the ability of the unencapsulated DP1004 strain to grow as sessile aggregates on collagen‐coated wells (Figure S1). These data indicate that collagen promotes the growth of pneumococci as surface‐adherent masses and that PfbB is at least partially involved in this process.

### 2.3 SSURE antibodies inhibit pneumococcal binding to collagens

**FIGURE 3:**
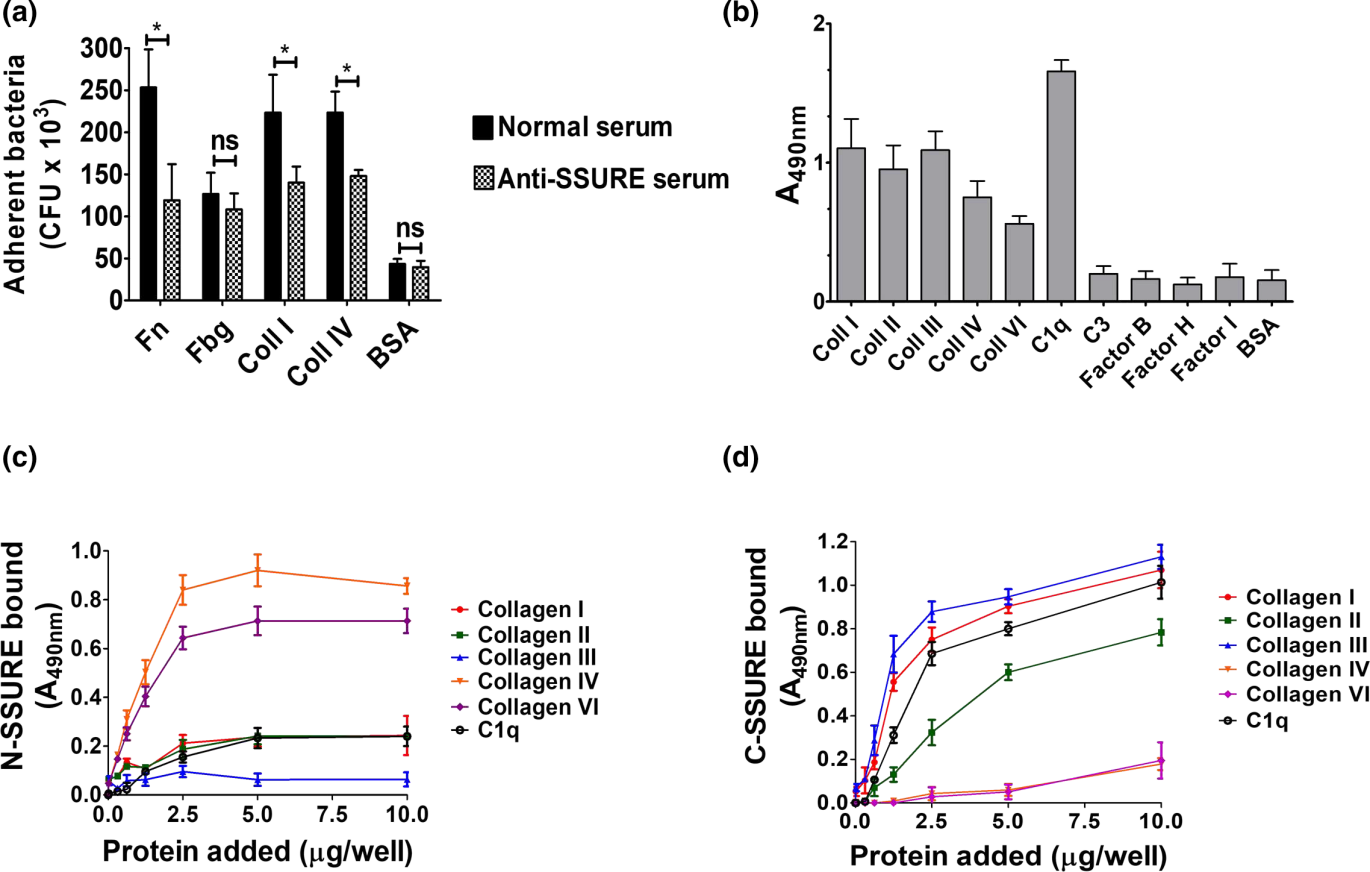
The C‐terminal PfbB SSURE domain binds to collagens and C1q. (a) Inhibition of binding of D39 pneumococci to collagens after bacterial pretreatment with serum raised in mice against recombinant PfbB C‐SSURE (anti‐SSURE serum). Serum from unimmunized mice (Normal serum) was used as a control. Bacterial binding was detected by counting colony‐forming units (CFU) after detachment of bacteria with trypsin. (b) Binding of histidine (his)‐tagged recombinant PfbB C‐SSURE to collagens and complement components. Binding was detected by ELISA using an anti‐his antibody. Plates were sensitized with 5 μg/ml of collagen types I, II, III, IV, VI, complement protein 1q (C1q), complement protein 3 (C3), factor B, factor H, factor I or bovine serum albumin (BSA), used as control. (c and d) Dose‐dependent binding of N‐SSURE (c) and C‐SSURE (d) domains to wells sensitized with 1 μg/ml of collagen type I, II, III, IV, VI and complement protein 1q (C1q). The binding of increasing concentrations of recombinant proteins fused to histidine (his) was revealed by ELISA using an anti‐his antibody. Shown are means ± SDs of three independent experiments conducted in triplicate.

The PfbB protein of the D39 strain contains six SSURE domains that contribute to 78% of the protein structure. Therefore, it was of interest to explore the effects of SSURE‐specific antibodies on bacterial adherence. To this end, we immunized mice with C‐SSURE, a recombinant PfbB fragment encompassing the C‐terminal SSURE domain (Figure 1a), which is >95% homologous to the four core repeats and tested the effects of immune sera on bacterial adherence. Pretreatment of encapsulated pneumococci with SSURE antibodies resulted in significant inhibition of their ability to bind to Fn as well as type I and IV collagen, but not to Fbg (Figure 3a). Collectively, these data suggest that the PfbB SSURE repeats are involved in the binding of pneumococci to collagen.

### 2.4 *S. pneumoniae* SSURE domains bind to collagen and C1q

Keeping in mind the ability of SSURE antibodies to inhibit *S. pneumoniae* adherence to collagen, we investigated whether SSURE domains directly bind to collagen. In a previous study, we found that two isolated PfbB fragments, each containing one SSURE domain, bound weakly to type I collagen (Papasergi et al., 2010). However, it could not be excluded from those data that the presence of a relatively bulky glutathione‐S‐transferase tag in the PfbB fragments interfered with such binding (Papasergi et al., 2010). Therefore, we produced two His‐tagged recombinant polypeptides encompassing the C‐terminal and the N‐terminal SSURE domains (C‐SSURE and N‐SSURE, respectively), of the PfbB protein (Figure 1a) and tested them for binding to collagens (types I, II, III, IV, and VI).

Since the complement system plays an important role in pneumococcal pathogenesis, we also tested the C‐SSURE fragment for binding to various complement factors (C3 and factors B, H, and I). In these experiments, plates were coated with human proteins and binding of C‐SSURE was detected by addition to the wells of a His tag antibody. Figure 3b shows that C‐SSURE is efficiently bound to collagen I, II, and III. Notably, C‐SSURE also efficiently bound C1q, but not the other complement components. To better define the binding activities of different SSURE domains, we tested graded doses of C‐SSURE and N‐SSURE, which differ significantly in amino acid sequence (Figure 1a), for their interactions with different types of collagens and C1q. Surprisingly, N‐SSURE is efficiently bound to type IV and VI, but not to type I, II, or III collagens or to C1q, while the opposite was true for C‐SSURE (Figure 3c,d). The binding of both recombinant fragments to their respective ligands was dose‐dependent and saturable. Data from these titration ELISA experiments were then used to calculate dissociation constants. C‐SSURE bound collagen I, III, and C1q with dissociation constants of, respectively, 1.72 ± 0.2, 1.16 ± 0.1, and 5.21 ± 0.4 μM, while N‐SSURE bound collagen IV and VI with dissociation constants of, respectively, 1.58 ± 0.1 and 1.20 ± 0.3 μM. Collectively, these data indicate that the two types of SSURE domains found in pneumococci bind to human collagens and C1q with an unexpected degree of specificity. C‐SSURE more efficiently recognizes fibrillar collagens, such as type I and III, as well as C1q compared with other collagen types. Conversely, N‐SSURE, which is only 73–75% identical to C‐SSURE, binds network‐forming collagens such as type IV and VI, but not fibrillar collagens or C1q.

### 2.5 Pneumococci bind to C1q via PfbB

**FIGURE 4:**
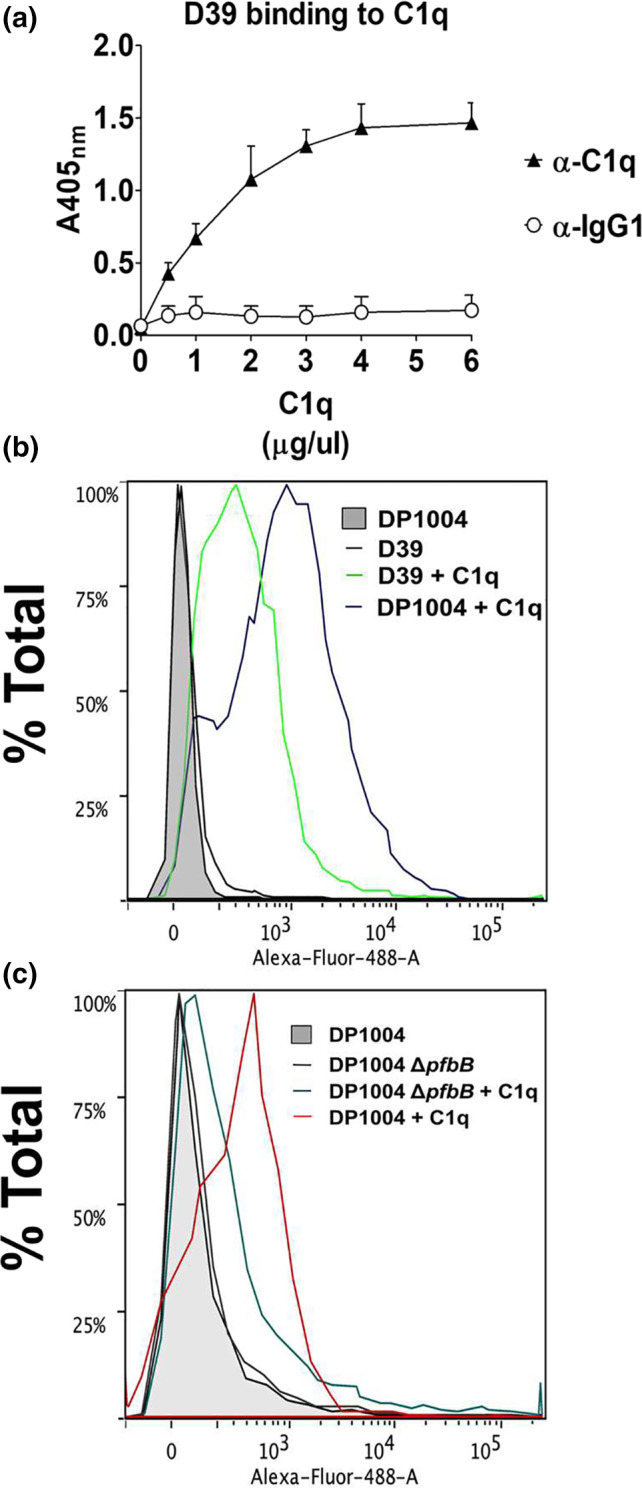
Role of PfbB in pneumococcal binding to C1q. The binding of C1q to pneumococci was measured by ELISA (a) or immunofluorescence flow cytometry (b and c). (a) Multi‐well plates sensitized with pneumococci (5 × 10^7^ CFU/ml) were incubated with increasing concentrations of C1q. Bound C1q was detected using anti‐C1q antibodies (α‐C1q). IgG1, isotype control. Shown are means ± SDs of three experiments performed in triplicate. (b) Binding of C1q (5 μg/ml) to encapsulated D39 (green line) and unencapsulated DP1004 (purple) strains as detected by immunofluorescence flow cytometry using an Alexa‐Fluor‐488‐labeled antibody. (c) Binding of C1q (5 μg/ml) to unencapsulated DP1004 (red) and its isogenic *pfbB* deletion mutant (Δ*pfbB*‐DP, dark green), as detected by immunofluorescence flow cytometry using an Alexa‐Fluor‐488‐labeled antibody.

In view of the ability of the C‐SSURE repeat to bind C1q, we assessed the contribution of the SSURE‐containing PfbB protein to the overall ability of pneumococci to bind C1q. As previously reported (Agarwal et al., 2013), we first observed that wild type D39 bacteria recognize and bind to C1q in a dose‐dependent manner (Figure 4a), and that unencapsulated bacteria were more efficient in C1q binding relative to the unencapsulated DP1004 strain (Figure 4b). Notably, the *ΔpfbB*‐DP deletion mutant was considerably impaired in C1q binding, with mean fluorescence intensity values that were considerably lower than those observed with the DP1004 unencapsulated parental strain (Figure 4c). All together, these data indicate that the PfbB protein plays a significant role in pneumococcal binding to C1q.

### 2.6 Role of PfbB in C1q‐mediated pneumococcal invasion of host cells

**FIGURE 5:**
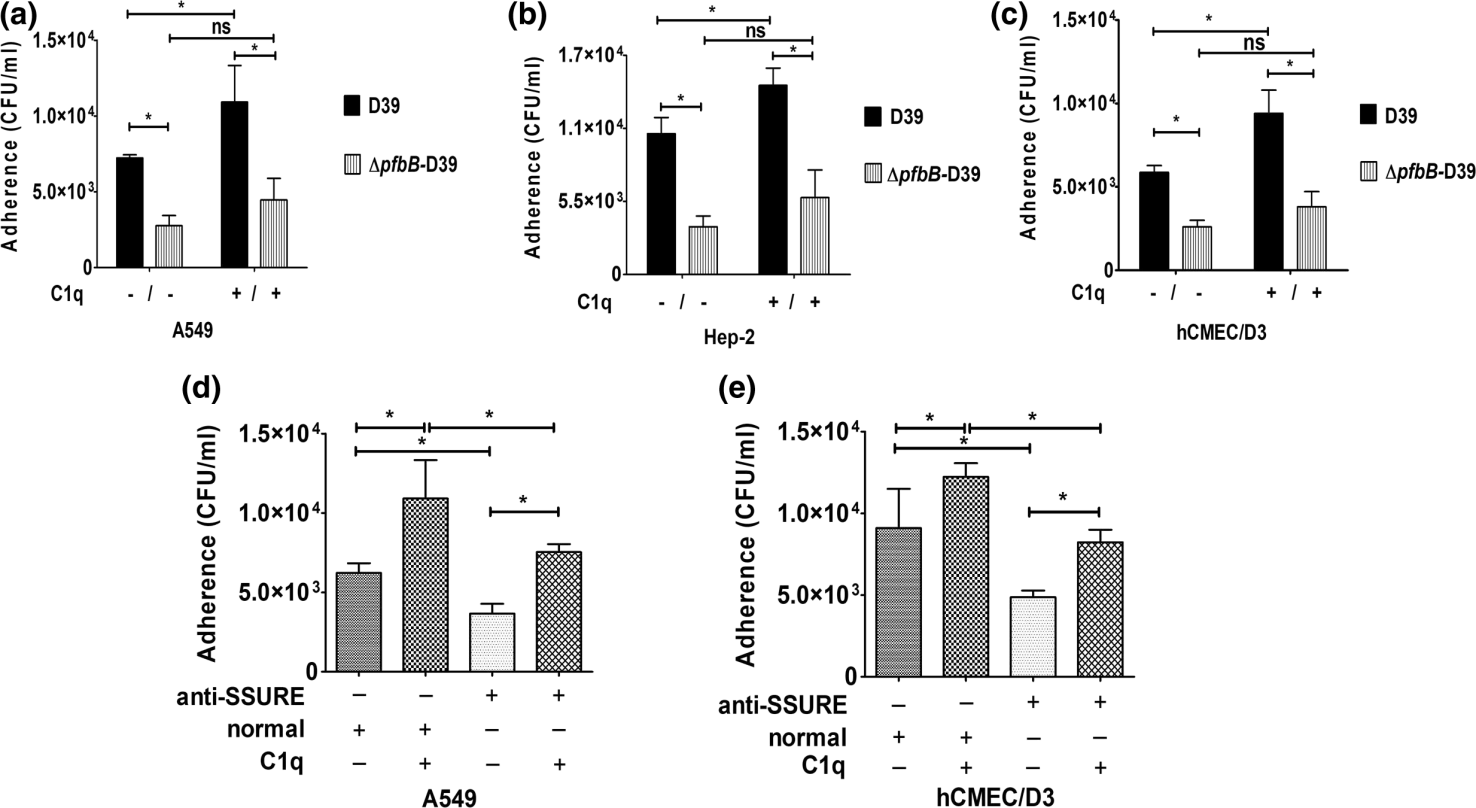
PfbB promotes C1q‐dependent adhesion to epithelial and endothelial cells. (a–c) monolayers of respiratory epithelial (A549, hep‐2) and brain microvascular endothelial (hCMEC/D3) cell lines were infected with pneumococcal strain D39 or its isogenic *pfbB* mutant in the presence (10 μg) or in the absence of C1q. Adhering bacteria were measured by colony‐forming units (CFU) counts. In some experiments (panels d and e) bacteria were pretreated with a pool of sera from animals immunized with C‐SSURE recombinant repeat (anti‐SSURE) or with a normal serum pool (normal). Shown are means ± SDs of three independent experiments conducted in triplicate. *p < 0.05 by Mann–Whitney test.

**FIGURE 6:**
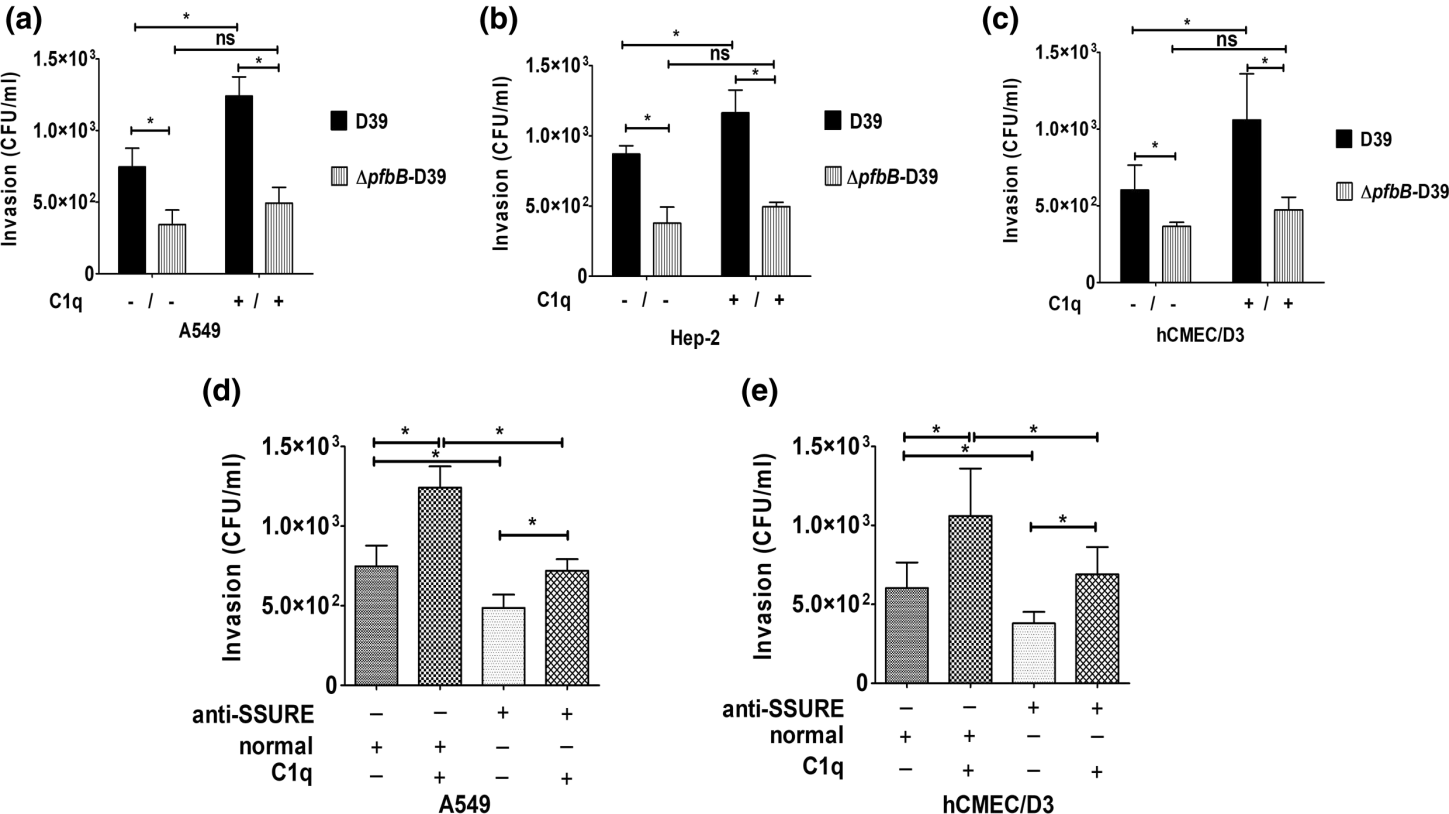
PfbB promotes C1q‐dependent invasion of epithelial and endothelial cells. (a–c) monolayers of respiratory epithelial (A549, hep‐2) and brain microvascular endothelial (hCMEC/D3) cell lines were infected with pneumococcal strain D39 or its isogenic *pfbB* mutant in the presence (10 μg) or the absence of C1q. Intracellular bacteria were measured by colony‐forming units (CFU) counts in an antibiotic protection assay. In some experiments (panels d and e) bacteria were pretreated with a pool of sera from animals immunized with the C‐SSURE recombinant domain (anti‐SSURE) or with a normal serum pool (normal). Shown are means ± SDs of three independent experiments conducted in triplicate. *p < 0.05 by Mann–Whitney test.

Pneumococcal adhesins may interact directly with host cell receptors or bind to host proteins that function as a bridge between the pathogen and host tissue. Previous studies have demonstrated that C1q bound to the pneumococcal surface promotes adherence to and invasion of host cells (Agarwal et al., 2013). Therefore, we investigated the role of the PfbB protein in promoting C1q‐mediated interactions between pneumococci and host cells. In these experiments, we used two human epithelial cell lines, namely A549 (lung) and Hep‐2 (larynx), as well as the brain microvascular endothelial cell line hCMEC/D3. In agreement with previous data (Agarwal et al., 2013), pretreatment of pneumococci with C1q considerably increased adherence of encapsulated wild type pneumococci to the cell lines tested (Figure 5a–c). Conversely, this effect was observed to a significantly lower extent or not at all using the *ΔpfbB*‐D39 mutant (Figure 5a–c) or when pneumococci were pretreated with SSURE antibodies (Figure 5d,e). When the role of the PfbB protein in promoting C1q‐mediated invasion was examined, the ability of encapsulated bacteria to invade microvascular endothelial cells as well as laryngeal or alveolar epithelial cells significantly increased in the presence of C1q. Interestingly, C1q was able to increase to similar degrees pneumococcal adhesion and invasion, indicating that C1q‐mediated adherence is followed by bacterial internalization (Figure 6). The invasion‐promoting activity of C1q was reduced in comparison with the parental strain when the *pfbB* mutant was tested (Figure 6a–c). A similar reduction of invasion was observed when pretreating wild type bacteria with C1q in the presence of SSURE antibodies (Figure 6d,e). Collectively, these data confirm previous studies indicating that, in the presence of C1q, pneumococci bind more efficiently to, and invade, epithelial and endothelial cells (Agarwal et al., 2014). Moreover, our data indicate that the ability of C1q to promote these interactions depends at least partially on the expression of PfbB on the bacterial surface.

### 2.7 C1q promotes pneumococcal adherence by binding to the α_2_β_1_ integrin

**FIGURE 7:**
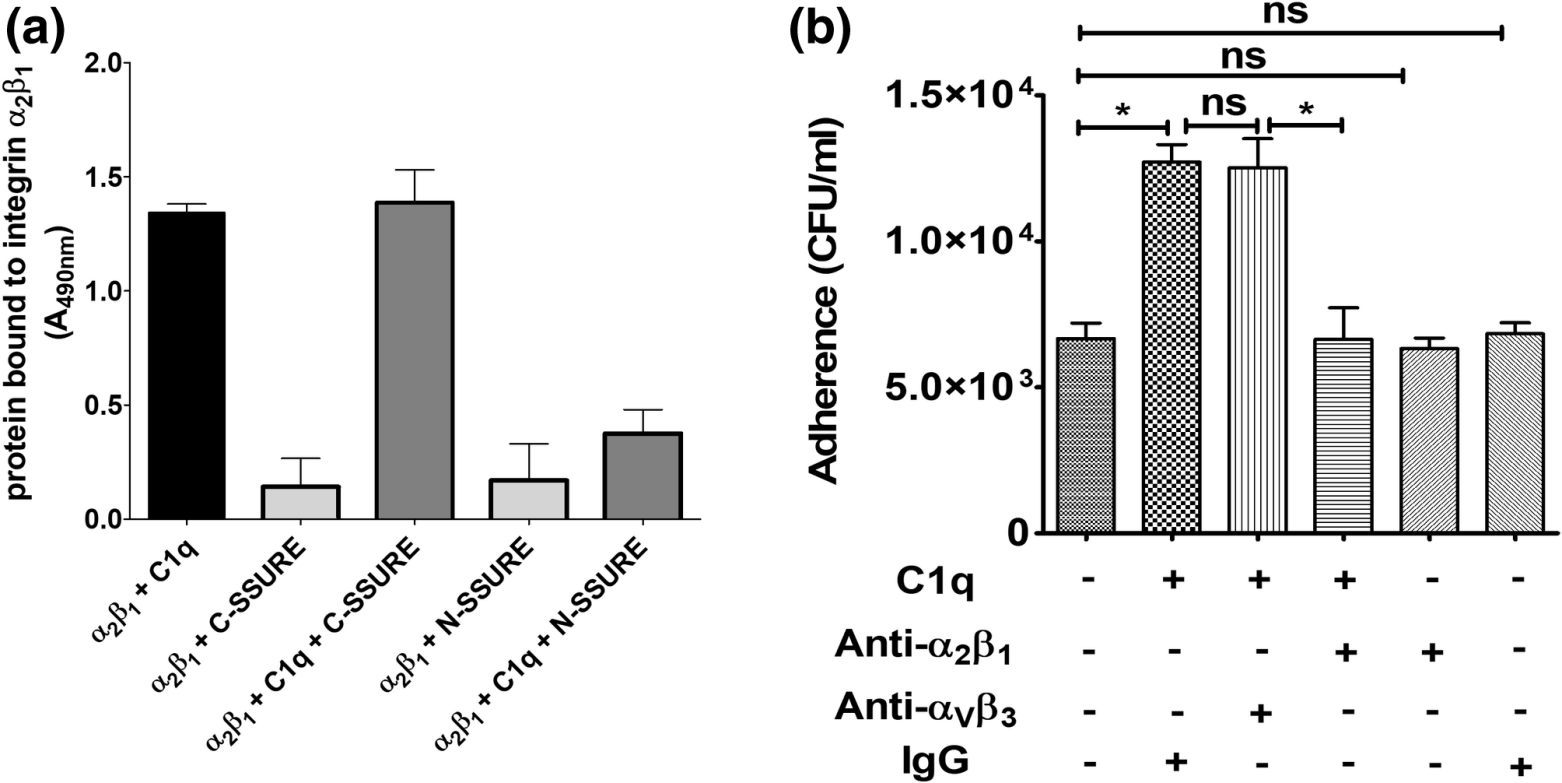
C1q acts as a bridge between the C‐SSURE domain and α_2_β_1_ integrin in mediating pneumococcal adhesion. (a) Interactions between C1q, recombinant SSURE domains (C‐SSURE or N‐SSURE), and α_2_β_1_‐integrin in ELISA assays. Binding was detected with anti‐C1q (black column) or anti SSURE antibodies. Shown are means ± SDs of three independent experiments conducted in triplicate. (b) Inhibitory effect of anti‐α_2_β_1_ antibodies on C1q‐mediated adhesion. Monolayers of respiratory epithelial cells (A549) were pretreated with antibodies against α_V_β_3_ or α_2_β_1_ integrins before being infected with bacteria in the presence (10 μg) or absence of C1q. Adhering bacteria were measured by colony‐forming units (CFU) counts. Shown are means ± SDs of three independent experiments conducted in triplicate. *p < 0.05 by Mann–Whitney test.

Previous studies have determined that the α_2_β_1_ integrin interacts with C1q and collagen via its I domain (Merle et al., 2021). By this mechanism, C1q acts as a bridge between *Bacillus anthracis* spores and α_2_β_1_ integrin expressed on the surface of respiratory cells to increase spore invasion of these cells (Xue et al., 2011). Therefore, it was of interest to ascertain whether C1q can also act as a bridge between the PfbB SSURE domains and the α_2_β_1_ integrin thereby promoting pneumococcal adherence. We first analyzed the interactions between C1q, isolated SSURE domains, and recombinant α_2_β_1_ integrin. C1q is readily bound to α_2_β_1_ integrin, as expected (Figure 7a). The further addition of C‐SSURE, but not N‐SSURE, resulted in the formation of a ternary complex with C1q and α_2_β_1_ integrin (Figure 7a). Next, we investigated the involvement of α_2_β_1_ in C1q‐mediated pneumococcal adherence using anti‐α_2_β_1_ antibodies. Figure 7b shows that pretreatment of epithelial cells with these antibodies, but not with control anti‐α_v_β_3_, markedly reduced C1q‐mediated adherence to A549 pneumocytes. Notably, anti‐α_2_β_1_ or IgG pretreatment did not affect bacterial adherence in the absence of C1q, indicating that these integrins do not function as direct receptors for pneumococci. Collectively, these data suggest that the ability of C1q to increase pneumococcal interactions with epithelial cells is at least partially related to the formation of a ternary complex involving the SSURE domains of PfbB, C1q, and α_2_β_1_ integrin.

## 3 DISCUSSION

Collagens are the body's most abundant proteins across the animal world and are likely to have played an important role in vertebrate evolution (Boot‐Handford & Tuckwell, 2003). Not surprisingly, symbiotic bacteria have co‐evolved various means to bind collagen, including the expression of specific cell‐wall adhesins that endow them with the ability to colonize a variety of tissues and cause persistent infections (Nobbs et al., 2009). Relatively little is known of the ability of pneumococci and their surface proteins to bind collagen. Investigations have focused on the classical striated fibrillar collagen type I with the exception of one study showing strong interactions of pneumococci with collagen type VI, which is abundantly expressed in the subepithelial surfaces of the upper and lower respiratory airways (Bober et al., 2010). However, pneumococcal proteins capable of binding collagen type VI have not been identified. In pneumococci, the presence of type‐1 pili has been associated with virulence and enhanced the ability to interact with epithelial and endothelial cells (Iovino et al., 2020). Proteins RrgA and RrgB were shown to be expressed along the entire pilus‐1 surface (Hilleringmann et al., 2008) and to consecutively interlock with collagen type I fibrils, as shown using atomic force microscopy (Becke et al., 2019). However, pilus‐1 is present in only 20–30% of pneumococcal clinical isolates.

We find here that SSURE domains from the PfbB protein, which is highly conserved among pneumococcal strains, can bind various types of collagen. Notably, we detected a considerable degree of selectivity in SSURE interactions with different collagen types. A recombinant fragment encompassing the C‐terminal repeat, which is >95% homologous to the core repeats, is bound more efficiently to fibril‐forming collagens (types I, II, and III) than to network‐forming collagens (type IV and VI). The latter was, in contrast, preferentially recognized by the N‐terminal repeat, which differs at least by 25% or more in amino acid sequence from the other repeats. Recognition of different collagen types by different SSURE domains, as shown here, seems to provide a good example of how bacteria acquire the ability to interact with an increasing number of ECM components by continuous duplication and variation of existing repeats rather than de novo protein creation. Moreover, repeat oligomerization can increase the capacity of bacteria to interact with host molecules. Indeed, although the collagen‐binding affinities of single SSURE domains are low (with dissociation constants in the micromolar range, as estimated here), the presence of multiple repeats is likely to increase PfbBB binding efficiency, as previously demonstrated for Fn and plasminogen binding (Jensch et al., 2010; Kanwal et al., 2017). Accordingly, PfbB deletion impaired the ability of encapsulated and unencapsulated pneumococci to interact with different collagen types, suggesting that this protein significantly contributes to the overall collagen‐binding activity of these bacteria.

SSURE domains are present not only in pneumococci but also in other pathogenic and commensal streptococcal species, such as *S. agalactiae* and *S. gordonii* (Buscetta et al., 2016). Since the ECM binding ability varies among SSURE domains from different streptococcal species, it will be of interest to investigate in future studies whether SSURE domains from *S. agalactiae* and *S. gordonii* also bind collagen and whether they display selectivity for different collagen types, as shown here for PfbB SSUREs. Although the PfbB protein plays a significant role in the ability of pneumococci to adhere to collagen, other proteins also seem to contribute to this activity as suggested by the residual binding activities of PfbB‐deletion mutants. DiiA is a highly conserved protein that efficiently binds to collagen type I (Escolano‐Martinez et al., 2016). Moreover, while in the present study, we used a non‐piliated reference strain, the RrgA and RrgB adhesins might make an important contribution to the ability of piliated stains to bind collagen, as mentioned above. Therefore, future studies should assess the relative contribution of PfbB, DiiA, and pilus‐1 to the ability of piliated and non‐piliated pneumococci to bind collagen. Currently available data suggest that adhesion of pneumococci to collagen involves the coordinated activity of different adhesins, in an analogous manner to adherence to fibrinogen of group B streptococci (Buscetta et al., 2014; Gutekunst et al., 2004; Papasergi et al., 2013). However, the structural basis of interaction with collagen of the above‐mentioned pneumococcal adhesins, which differ in sequence and structural organization, remains to be determined.

Well‐organized pneumococcal biofilms are frequently found in the nasopharynx of individuals with persistent pneumococcal carriage. Moreover, the propensity of single strains to form biofilms is inversely related to the amount of capsular material (Chao et al., 2015). Accordingly, we found here that the encapsulated D39 strain has little ability to grow as surface‐adherent aggregates on plastic surfaces. This strain, however, did grow in the sessile form on collagen‐coated wells, in a manner that was partially dependent on the expression of the PfbB protein. It will be of interest to determine whether sessile growth on collagen, as described here, has common features with in vivo biofilm formation (Chao et al., 2015) and with in vitro biofilm models that depend on the presence of competence stimulating peptide and its receptor (Oggioni et al., 2006). It will also be of interest to assess whether PfbB expression is increased in biofilms and promotes in vivo biofilm formation.

Our study shows that SSURE repeats from *S. pneumoniae* also bind C1q, the first component of the classical complement pathway. This is reminiscent of collagen‐binding proteins from other bacterial pathogens. Collagen adhesin (Cna) from *Staphylococcus aureus* (Valotteau et al., 2017) and Cna‐like proteins make up a large family of structurally related virulence factors from Gram‐positive (Kreikemeyer et al., 2005; Krishnan & Narayana, 2011; Patti & Hook, 1994; Sillanpaa et al., 2009; Xu et al., 2004) and Gram‐negative (Fink et al., 2002; MacKichan et al., 2008; Patti et al., 1994; Wagner et al., 2007) bacteria. These proteins bind both collagen and C1q and inhibit the classical complement activation pathway (Kang et al., 2013). Interestingly, Cna‐like proteins are found in streptococcal species, such as *Streptococcus mutans*, *Streptococcus equi*, *Enterococcus faecalis*, and *Enterococcus faecium*, that do not possess SSURE‐containing proteins (Kang et al., 2013; Sillanpaa et al., 2009). Studies are underway to ascertain whether SSURE repeats from *S. gordonii*, *S. mitis*, and *S. agalactiae* bind C1q and inhibit, by these means, the classical complement activation pathway.

In the present study, we focused on another recently described functional feature of C1q, namely its ability to promote pneumococcal adherence to and invasion of host cells. Notably, functionally active complement components have been detected in airway secretions (Sarma et al., 2006). In this scenario, pneumococci can recruit C1q on their surface and use it as a bridge to interact with epithelial and endothelial cells (Agarwal et al., 2013; Agarwal et al., 2014). In a study performed by Xu et al. (2004), it was found that *Bacillus anthracis* spores interact via the LPXTG surface protein BclA with C1q, which in turn binds to the α_2_β_1_ integrin on host cells and promotes by this mechanism spore entry into epithelial cells (Xue et al., 2011). Thus, it was of interest to ascertain whether a similar mechanism is involved in the pneumococcal invasion of respiratory epithelial cells. This seems to be the case, as shown here by the formation of a ternary complex comprising C‐SSURE, C1q, and α_2_β_1_. SSURE domains were, however, unable to directly interact with this integrin. Moreover, pretreatment of respiratory epithelial cells with anti‐α_2_β_1_ largely prevented C1q‐dependent, but not C1q‐independent, pneumococcal adherence. These data indicate that, albeit not a primary or direct receptor for pneumococci, α_2_β_1_ integrin is involved in adherence and subsequent entry of these bacteria in the presence of C1q. Therefore, our data add pneumococci to the growing list of pathogens that target α_2_β_1_ integrin for entry into epithelial cells, including group A streptococci, *B. anthracis*, echovirus 1, and rotavirus (Bergelson et al., 1992; Caswell et al., 2007; Ciarlet et al., 2002; Xue et al., 2011). Our data do not exclude, of course, the possibility that C1q bound to the surface of pneumococci interacts with receptors other than α_2_β_1_, such as CD91 and gC1q‐R (receptor for the globular head of C1q; Ogden et al., 2001), a well‐characterized bacterial internalization receptor (Braun et al., 2000). Finally, it should be noted that other proteins, such as the pneumococcal PepO protein that also binds C1q, can participate in C1q‐mediated interactions with host cells (Agarwal et al., 2014). This is notion is supported by our data showing that anti‐SSURE antibodies were unable to completely block C1q‐mediated adherence and internalization.

In conclusion, our data indicate an important role of the PfbB SSURE domains in promoting interactions of pneumococci with collagens, C1q and host cells. Since the colonization of epithelial cells and invasion of collagen‐rich subepithelial tissues are essential steps in pneumococcal pathogenesis, these data may be useful to devise alternative strategies to control infections caused by antibiotic‐resistant strains.

## 4 MATERIALS AND METHODS

### 4.1 Bacterial strains and reagents

The encapsulated strain D39, serotype 2, (Iannelli et al., 1999; Shoemaker & Guild, 1974), and its unencapsulated DP1004 derivative (Iannelli et al., 2004) were used in this study. Deletion of the *pfbB* gene was accomplished by transformation of D39 or DP1004 strains, as described previously (Papasergi et al., 2010), yielding strains *ΔpfbB‐*D39, and *ΔpfbB‐*DP. All bacteria were grown in Todd‐Hewitt broth (Difco Laboratories) supplemented with 0.5% yeast extract (THY, Thermo Fisher Scientific) at 37°C in 5% CO_2_. We used in this study human fibronectin (Sigma‐Aldrich; F0895), human fibrinogen (Fluka Analytical; 46,313), and C1q (Calbiochem; 204,876). Type I and III collagens from calfskin were generously donated by Dr R. Tenni, Department of Molecular Medicine, University of Pavia, Italy. Collagen type II was prepared from bovine nasal cartilage according to Stawich and Nimmi (Strawich & Nimni, 1971). Human type IV and VI collagens were from Sigma‐Aldrich. Complement Factors B, H, and I were purchased from R&D (Minneapolis MN, USA).

### 4.2 Recombinant PfbB SSURE fragment and antisera

In the present study, we used two recombinant fragments designated C‐SSURE and N‐SSURE encompassing the C‐terminal and N‐terminal SSURE domains of the PfbB protein, respectively. C‐SSURE is a recombinant polyhistidine‐tagged polypeptide encompassing amino acids 902–1053 of the PfbB protein, while N‐SSURE is an identically tagged polypeptide encompassing amino acids 144–293 (Figure 1a). The encoding DNA fragments were cloned from genomic *Streptococcus pneumoniae* D39 DNA into the pET21b vector (Thermo Fisher Scientific, Milan, Italy). The C‐SSURE encoding genomic fragment was amplified using the primers c‐ter_up: 5′‐AAACATATGGGTTTAATTTCTAAAGAAACTGTCGAAAAAG‐3′ and c‐ter_low: 5′‐AGTGCTCGAGTTCTTTAACATTTATCTTAATAG‐3′. The N‐SSURE encoding genomic fragment was amplified using the primers N‐ter_up: 5′‐AAACATATGGCAGAAACTACTCCTGAAC‐3′ and N‐ter_low: 5′‐AGTGCTCGAGATTGATGTTGATGTCTACATT‐3′. Both fragments were cloned into the *Xho*I and *Nhe*I restriction sites of pET21b. The recombinant plasmids were used to transform *E. coli* BL21(DE3) bacteria that were grown in the Luria Bertani medium (LB; Life Science) at 37°C with ampicillin (100 μg/ml) until their mid‐log phase (OD_560nm_ = 0.6). Next, the bacteria were induced with 1 mM isopropyl‐β‐D‐thiogalactopyranoside (IPTG; Sigma‐Aldrich; I6758) under shaking for 18 h at 30°C. The bacteria were then washed with phosphate‐buffered saline (PBS: 137 mM NaCl; 2,7 mM KCl; 10 mM Na_2_HPO_4_; 1,8 mM KH_2_PO_4_), resuspended in lysis buffer (PBS; 100 μg/ml lysozyme, Fluka Analytical, 62,970; 25 mM imidazole, Sigma‐Aldrich, I5513; 1 tablet per 50 ml of complete, EDTA‐free protease inhibitor cocktail, Roche Diagnostics, 11,873,580,001) and lysed using a sonicator (MSE Soniprep 150, 4 1‐min bursts). Both recombinant SSURE fragments were purified from lysate supernatants using a nickel affinity column (HisTrap HP; GE Healthcare, Italy). The purity of these preparations was assessed by polyacrylamide gel electrophoresis (Figure S2a). Anti‐SSURE serum was produced by immunizing 6‐week‐old specific pathogen‐free CD1 mice (Charles River Laboratories, Italia) by the intraperitoneal injection of 50 μg of the C‐SSURE fragment in alum on days 0, 14, and 28. Sera were collected 15 days after the last immunization. The PfbB‐specificity of SSURE sera was verified in ELISA assays by observing their lack of reactivity against bacteria deleted for *pfbB* (Figure S2b). To obtain anti‐pneumococcus serum, mice were immunized using 50 μg of a crude pneumococcal extract obtained as previously described (Papasergi et al., 2010). Immunizations were conducted according to the European Union guidelines for the handling of laboratory animals and to the Italian law, as detailed below under “Ethics Approval.”

### 4.3 Bacterial binding to immobilized host proteins

Bacterial adhesion to extracellular matrix components was determined by ELISA assays. For this purpose, 96 well of Nunc MaxiSorp flat‐bottom plates (Thermo Fisher Scientific; 44‐2404‐21) were sensitized at 4°C overnight with different substrates (5 μg/well) dissolved in 0.05 M carbonate buffer (pH 9.5). After blocking with 5% BSA in Tris‐Buffered Saline pH 7.5 (TBS; 50 mM Tris‐Cl; 150 mM NaCl), pneumococci (10^7^/well) were added to the plates and incubated for 2 h at room temperature (RT). After washing, pneumococcal binding to the wells was detected with anti‐pneumococcus serum diluted 1:500 in TBS‐1% milk and incubated for an additional 1 h at 37°C. To detect antibody binding, anti‐mouse IgG conjugated with alkaline phosphatase (Sigma‐Aldrich; A7434) diluted 1:2000 in TBS‐1% milk was added and left for 1 h at 37°C. After the addition of para‐phenyl phosphate, absorbance at 405 nm was determined in an ELISA plate reader. In some experiments, bacterial attachment to host extracellular matrix components was assessed by colony‐forming units (CFU) counting adherent pneumococcal cells. To this end, pneumococci were preincubated for 15 min at 37°C with sera and then added to substrate‐coated‐wells and incubated for 1 h at 37°C in 5% CO_2_. After extensive washing, the wells were treated with trypsin (2.5 mg/ml; Sigma‐Aldrich) for 10 min at 37°C in 5% CO_2_ to release the attached bacteria, which were enumerated on agar plates.

### 4.4 Growth on collagen‐coated surfaces

Pneumococci were grown in THY broth to the early exponential phase (OD_600nm_ ~ 0.3), diluted 1:5 in fresh THY medium, and added to 6‐well polystyrene flat‐bottom plates that had been coated with human collagen type I (5 μg/ml) or BSA (5 μg/ml), used as control. Plates were incubated at 37°C in 5% CO_2_ for the following 16 h. Unattached bacteria were then gently decanted from the wells that were carefully washed with PBS (137 mM NaCl; 2,7 mM KCl; 10 mM Na_2_HPO_4_, 1,8 mM KH_2_PO_4_) and air‐dried. Adherent bacteria were stained for 15 min with a 0.1% (w/v) solution of crystal violet (Sigma‐Aldrich). After rinsing with PBS, the bound dye was released from stained cells using ethanol/acetone 80:20 (vol/vol) and quantified by measuring A_595 nm_ values.

### 4.5 C‐SSURE binding to human collagens and complement factors

To test the binding of the recombinant C‐SSURE and N‐SSURE fragments to immobilized substrates, wells were coated overnight at 4 °C in 0.1 M sodium carbonate, pH 9.5, with the indicated concentrations of human collagens or complement factors. The plates were washed three times with 0.5% (v/v) Tween 20 in PBS. To block additional protein binding sites, the wells were treated for 1 h at 22 °C with 2% (v/v) BSA in PBS. Increasing concentrations of C‐SSURE or N‐SSURE (0,016–10 μg/ml) were then added to the wells. Binding was detected by the addition of a mouse monoclonal antibody directed against the His‐tag (Sigma‐Aldrich) in 1% BSA‐PBS for 1 h at RT, followed by a 45 min incubation with anti‐mouse IgG horseradish peroxidase‐conjugated antibody. Absorbance at 490 nm was determined after the addition of 3, 3′, 5, 5′‐tetramethylbenzidine or o‐phenylenediamine dihydrochloride (Sigma‐Aldrich) as a substrate.

### 4.6 Complex formation with α_2_β_1_ integrin

The binding of 1 μg of C1q to surface coated α_2_β_1_ integrin (R&D systems; 250 ng/well) was detected by the addition of rabbit anti‐human C1q IgG (1:1000) followed by HRP‐conjugated IgG secondary antibodies as described above. Binding of SSURE fragments to the integrin was detected using mouse monoclonal anti‐His IgG (1:1000) followed by rabbit HRP‐conjugated anti‐mouse IgG, as described above. To assess ternary complex formation between α_2_β_1_ integrin, C1q, and SSURE domains, microtiter wells coated with 250 ng of α_2_β_1_ integrin were incubated with 1 μg of C1q for 1 h followed by incubation with 1 μg of either C‐SSURE or N‐SSURE. After 1 h, the wells were incubated with a mouse monoclonal anti‐His IgG followed by rabbit HRP‐conjugated anti‐mouse IgG as described above.

### 4.7 Analysis of C1q binding to bacteria

To investigate the binding of C1q to pneumococci, bacteria were grown in THY broth to the early exponential phase (OD_600nm_ ~ 0.3), washed in TBS, and distributed in 96‐well plates (5 × 10^7^ CFU/well). Afterward, wells were blocked with TBS‐2% milk and incubated with the indicated doses of human recombinant C1q (Calbiochem; 204,876) for 1 h at 37°C. Cell‐surface bound C1q was revealed with a mouse anti‐C1q monoclonal antibody (Abcam; ab71940) diluted 1:100 in TBS‐1% milk followed by incubation with AP‐conjugated anti‐mouse IgG (Sigma‐Aldrich; A7434).

For flow cytometry immunofluorescence analysis, bacteria were incubated with recombinant human C1q (5 μg/ml) for 1 h at room temperature in constant agitation. Washed bacteria were incubated with anti‐C1q antibody (Abcam; ab71940) diluted 1:100 in 1% milk‐PBS for 1 h at room temperature in constant agitation. Afterward, to detect the binding of C1q to the bacteria, an Alexa‐Fluor‐488‐labeled anti‐mouse antibody was used. Bacteria were analyzed with a Flow Cytometer (BD FACS canto II) using the Flowlogic software.

### 4.8 Bacterial adhesion and invasion assays

The human cell lines A549 (type II alveolar epithelial cells, ATCC CCL‐185), HEp‐2 (laryngeal epithelial cells, ATCC CCL‐23), and hCMEC/D3 (brain microvascular endothelial cells, kindly provided by P.O. Couraud, INSERM, Paris, France) were grown in, respectively, F‐12 medium (ATCC 30–2004) and Eagle's Minimum Essential Medium (ATCC 30–2003) with 10% (vol/vol) fetal bovine serum (1203C, Sigma‐Aldrich) and Endothelial Cell Medium 2 (C‐22011, Promo Cell), supplemented with SupplementMix (C‐39216, Promo Cell), at 37 °C in a humidified 5% CO_2_ incubator.

For adherence and invasion assays, cells were dispensed into 12‐well plates at a density of 5 × 10^4^/well and grown in 5% CO_2_ for 48 h before the assay. The monolayers were then washed three times with Dulbecco's PBS (DPBS; Euroclone) to remove any residual medium. Bacteria were grown to the early log phase (OD_600nm_ ~ 0.3), washed, resuspended in RPMI without FBS, and applied to the monolayers at a multiplicity of infection of 30. For adherence assays, infected monolayers were incubated for 1 h at 37°C in 5% CO_2_ and washed three times to remove non‐adherent bacteria. In preliminary experiments, we determined that, under these conditions, there is no significant bacterial internalization. For studying C1q‐mediated pneumococcal adhesion, bacteria were incubated for 20 min with 10 μg human recombinant C1q in a total volume of 100 μl at 37°C in 5% CO_2_ prior to addition to the monolayers. Post‐infection, cells were washed three times with DPBS to remove unbound bacteria. After the addition of cold H_2_O and scraping, cell lysates were serially diluted and plated onto agar plates for colony counting. For invasion assays, after 1 h of adhesion, monolayers were washed and further incubated for 1 h with a medium containing bactericidal amounts of penicillin and streptomycin (200 U/ml and 200 μg/ml, respectively), as previously described. Adherence and invasion values were expressed as the number of cell‐associated or invading bacteria. Blocking antibodies against integrin α_2_β_1_ and α_ν_β_3_ and control non‐immune antibodies were obtained in rabbits as previously described (De Gaetano et al., 2018). To assess the effects of blocking antibodies on pneumococcal adherence, cells were pre‐incubated for 1 h with the appropriate antibodies diluted 1:1000 in a cell culture medium and then incubated with bacteria.

### 4.9 SSURE immune sera

To obtain sera, mice were immunized at the animal facilities of the Department of Pathology of the University of Messina, according to the European Union guidelines for the handling of laboratory animals. The procedure was approved by the local animal experimentation committee (OPBA) and by national authorities (Ministero della Salute, permit no.‐785/2018‐PR).

### 4.10 Statistical analysis

All experiments were repeated at least three times and the data were expressed as means ± standard deviations (SD). Data were analyzed for statistical significance by the Mann–Whitney test. A p‐value of 0.05 was used as the threshold for significance.

## AUTHOR CONTRIBUTIONS

Concetta Beninati, Pietro Speziale, Giuseppe Teti and Giampiero Pietrocola conceived the study. Giuseppe Valerio De Gaetano, Giampiero Pietrocola, Germana Lentini, Agata Famà, Concetta Beninati and Giuseppe Teti designed the experiments. Giuseppe Valerio De Gaetano, Germana Lentini, Agata Famà, Chiara Cullotta, Ivana Raffaele, Francesco Coppolino and Chiara Motta carried out the experiments and analyzed the data. Concetta Beninati, Giuseppe Teti and Giuseppe Valerio De Gaetano wrote the paper. All authors gave approval to the final version of the paper.

## CONFLICT OF INTEREST

Concetta Beninati and Giuseppe Teti act as scientific advisors for, respectively, Scylla Biotech Srl. and Charybdis Vaccines Srl. without receiving any compensation for these activities. Charybdis Vaccines S.r.l. and Scylla Biotech S.r.l. did not provide funding for this study and had no role in its conduction. The remaining authors declare that the research was conducted in the absence of any commercial or financial relationships that could be construed as a potential conflict of interest.

## ETHICS APPROVAL

The procedure for obtaining serum samples from immunized animals was approved by the local animal experimentation committee (OPBA) and by the relevant national authority (Ministero della Salute, permit no.‐785/2018‐PR). Human samples, except for cell lines, were not used in this study.

## Supporting information


Figure S1

Figure S2

FigureCaption

## Data Availability

The data that support the findings of this study are available from the corresponding author upon reasonable request.

## References

[mmi14920-bib-0001] Agarwal, V. , Ahl, J. , Riesbeck, K. & Blom, A.M. (2013) An alternative role of C1q in bacterial infections: facilitating *Streptococcus pneumoniae* adherence and invasion of host cells. Journal of Immunology, 191, 4235–4245. 10.4049/jimmunol.1300279 24038089

[mmi14920-bib-0002] Agarwal, V. & Blom, A.M. (2015) Roles of complement C1q in pneumococcus‐host interactions. Critical Reviews in Immunology, 35, 173–184. 10.1615/critrevimmunol.2015012177 26559226

[mmi14920-bib-0003] Agarwal, V. , Sroka, M. , Fulde, M. , Bergmann, S. , Riesbeck, K. & Blom, A.M. (2014) Binding of *Streptococcus pneumoniae* endopeptidase O (PepO) to complement component C1q modulates the complement attack and promotes host cell adherence. Journal of Biological Chemistry, 289, 15833–15844. 10.1074/jbc.M113.530212 24739385 PMC4140937

[mmi14920-bib-0004] Arora, S. , Gordon, J. & Hook, M. (2021) Collagen binding proteins of gram‐positive pathogens. Frontiers in Microbiology, 12, 628798. 10.3389/fmicb.2021.628798 33613497 PMC7893114

[mmi14920-bib-0005] Becke, T.D. , Ness, S. , Kaufmann, B.K. , Hartmann, B. , Schilling, A.F. , Sudhop, S. et al. (2019) Pilus‐1 backbone protein RrgB of *Streptococcus pneumoniae* binds collagen I in a force‐dependent way. ACS Nano, 13, 7155–7165. 10.1021/acsnano.9b02587 31184856

[mmi14920-bib-0006] Beghetto, E. , Gargano, N. , Ricci, S. , Garufi, G. , Peppoloni, S. , Montagnani, F. et al. (2006) Discovery of novel *Streptococcus pneumoniae* antigens by screening a whole‐genome lambda‐display library. FEMS Microbiology Letters, 262, 14–21. 10.1111/j.1574-6968.2006.00360.x 16907734

[mmi14920-bib-0007] Bella, J. & Hulmes, D.J. (2017) Fibrillar Collagens. Subcell Biochem, 82, 457–490. 10.1007/978-3-319-49674-0_14 28101870

[mmi14920-bib-0008] Bergelson, J.M. , Shepley, M.P. , Chan, B.M.C. , Hemler, M.E. & Finberg, R.W. (1992) Identification of the integrin Vla‐2 as a receptor for Echovirus‐1. Science, 255, 1718–1720. 10.1126/science.1553561 1553561

[mmi14920-bib-0009] Bingham, R.J. , Rudino‐Pinera, E. , Meenan, N.A.G. , Schwarz‐Linek, U. , Turkenburg, J.P. , Hook, M. et al. (2008) Crystal structures of fibronectin‐binding sites from *Staphylococcus aureus* FnBPA in complex with fibronectin domains. Proceedings of the National Academy of Sciences of the United States of America, 105, 12254–12258. 10.1073/pnas.0803556105 18713862 PMC2518095

[mmi14920-bib-0010] Binsker, U. , Kohler, T.P. , Krauel, K. , Kohler, S. , Habermeyer, J. , Schwertz, H. et al. (2017) Serotype 3 pneumococci sequester platelet‐derived human thrombospondin‐1 via the adhesin and immune evasion protein hic. Journal of Biological Chemistry, 292, 5770–5783. 10.1074/jbc.M114.623876 28209711 PMC5392572

[mmi14920-bib-0011] Binsker, U. , Kohler, T.P. , Krauel, K. , Kohler, S. , Schwertz, H. & Hammerschmidt, S. (2015) Pneumococcal adhesins PavB and PspC are important for the interplay with human thrombospondin‐1. Journal of Biological Chemistry, 290, 14542–14555. 10.1074/jbc.M114.623876 25897078 PMC4505522

[mmi14920-bib-0012] Bober, M. , Enochsson, C. , Collin, M. & Morgelin, M. (2010) Collagen VI is a subepithelial adhesive target for human respiratory tract pathogens. Journal of Innate Immunity, 2, 160–166. 10.1159/000232587 20375633

[mmi14920-bib-0013] Bogaert, D. , De Groot, R. & Hermans, P.W.M. (2004) *Streptococcus pneumoniae* colonisation: the key to pneumococcal disease. Lancet Infectious Diseases, 4, 144–154. 10.1016/S1473-3099(04)00938-7 14998500

[mmi14920-bib-0014] Boot‐Handford, R.P. & Tuckwell, D.S. (2003) Fibrillar collagen: the key to vertebrate evolution? A tale of molecular incest. Bioessays, 25, 142–151. 10.1002/bies.10230 12539240

[mmi14920-bib-0015] Braun, L. , Ghebrehiwet, B. & Cossart, P. (2000) gC1q‐R/p32, a C1q‐binding protein, is a receptor for the InlB invasion protein of listeria monocytogenes. Embo Journal, 19, 1458–1466. 10.1093/emboj/19.7.1458 10747014 PMC310215

[mmi14920-bib-0016] Bumbaca, D. , Littlejohn, J.E. , Nayakanti, H. , Rigden, D.J. , Galperin, M.Y. & Jedrzejas, M.J. (2004) Sequence analysis and characterization of a novel fibronectin‐binding repeat domain from the surface of Streptococcus pneumoniae. Omics‐a Journal of Integrative Biology, 8, 341–356. 10.1089/omi.2004.8.341 15703481

[mmi14920-bib-0017] Buscetta, M. , Firon, A. , Pietrocola, G. , Biondo, C. , Mancuso, G. , Midiri, A. et al. (2016) PbsP, a cell wall‐anchored protein that binds plasminogen to promote hematogenous dissemination of group B streptococcus. Molecular Microbiology, 101, 27–41. 10.1111/mmi.13357 26888569

[mmi14920-bib-0018] Buscetta, M. , Papasergi, S. , Firon, A. , Pietrocola, G. , Biondo, C. , Mancuso, G. et al. (2014) FbsC, a novel fibrinogen‐binding protein, promotes *Streptococcus agalactiae*‐host cell interactions. Journal of Biological Chemistry, 289, 21003–21015. 10.1074/jbc.M114.553073 24904056 PMC4110306

[mmi14920-bib-0019] Caswell, C. , Lukomska, E. , Seo, N.S. , Höök, M. & Lukomski, S. (2007) Scl1‐dependent internalization of group a streptococcus via direct interactions with the alpha2beta(1) integrin enhances pathogen survival and re‐emergence. Mol Microbiol., 64(5), 1319–1331. 10.1111/j.1365-2958.2007.05741.x 17542923

[mmi14920-bib-0020] Chao, Y.S. , Marks, L.R. , Pettigrew, M.M. & Hakansson, A.P. (2015) *Streptococcus pneumoniae* biofilm formation and dispersion during colonization and disease. Frontiers in Cellular and Infection Microbiology, 4, 194. 10.3389/fcimb.2014.00194 25629011 PMC4292784

[mmi14920-bib-0021] Ciarlet, M. , Crawford, S. , Cheng, E. , Blutt, S. , Rice, D. , Bergelson, J.M. et al. (2002) VLA‐2 (alpha2beta1) integrin promotes rotavirus entry into cells but is not necessary for rotavirus attachment. Journal of Virology, 76(3), 1109–1123. 10.1128/JVI.76.3.1109-1123.2002 11773387 PMC135817

[mmi14920-bib-0022] De Gaetano, G.V. , Pietrocola, G. , Romeo, L. , Galbo, R. , Lentini, G. , Giardina, M. et al. (2018) The *Streptococcus agalactiae* cell wall‐anchored protein PbsP mediates adhesion to and invasion of epithelial cells by exploiting the host vitronectin/α(v) integrin axis. Molecular Microbiology, 110, 82–94. 10.1111/mmi.14084 30030946

[mmi14920-bib-0023] Domenech, M. , Garcia., E. & Moscoso, M. (2012) Biofilm formation in *Streptococcus pneumoniae* . Microbial Biotechnology, 5(4), 455–465. 10.1111/j.1751-7915.2011.00294.x 21906265 PMC3815323

[mmi14920-bib-0024] Escolano‐Martinez, M.S. , Domenech, A. , Yuste, J. , Cercenado, M.I. , Ardanuy, C. , Linares, J. et al. (2016) DiiA is a novel dimorphic cell wall protein of *Streptococcus pneumoniae* involved in invasive disease. Journal of Infection, 73, 71–81. 10.1016/j.jinf.2016.04.010 27105656

[mmi14920-bib-0025] Fink, D.L. , Green, B.A. & St. Geme, J.W. (2002) The *Haemophilus influenzae* hap autotransporter binds to fibronectin, laminin, and collagen IV. Infection and Immunity, 70, 4902–4907. 10.1128/IAI.70.9.4902-4907.2002 12183535 PMC128251

[mmi14920-bib-0026] Gutekunst, H. , Eikmanns, B.J. & Reinscheid, D.J. (2004) The novel fibrinogen‐binding protein FbsB promotes *Streptococcus agalactiae* invasion into epithelial cells. Infection and Immunity, 72, 3495–3504. 10.1128/IAI.72.6.3495-3504.2004 15155657 PMC415667

[mmi14920-bib-0027] Hammerschmidt, S. , Rohde, M. & Preissner, K.T. (2019) Extracellular matrix interactions with gram‐positive pathogens. Microbiology Spectrum, 7(2), 1–21. 10.1128/microbiolspec.GPP3-0041-2018 PMC11590433 31004421

[mmi14920-bib-0028] Hilleringmann, M. , Giusti, F. , Baudner, B.C. , Masignani, V. , Covacci, A. , Rappuoli, R. et al. (2008) Pneumococcal pili are composed of protofilaments exposing adhesive clusters of Rrg A. PLoS Pathogens, 4(3), e1000026. 10.1371/journal.ppat.1000026 18369475 PMC2265430

[mmi14920-bib-0029] Holmes, A.R. , McNab, R. , Millsap, K.W. , Rohde, M. , Hammerschmidt, S. , Mawdsley, J.L. et al. (2001) The pavA gene of *Streptococcus pneumoniae* encodes a fibronectin‐binding protein that is essential for virulence. Molecular Microbiology, 41, 1395–1408. 10.1046/j.1365-2958.2001.02610.x 11580843

[mmi14920-bib-0030] Iannelli, F. , Chiavolini, D. , Ricci, S. , Oggioni, M.R. & Pozzi, G. (2004) Pneumococcal surface protein C contributes to sepsis caused by *Streptococcus pneumoniae* in mice. Infection and Immunity, 72, 3077–3080. 10.1128/IAI.72.5.3077-3080.2004 15102826 PMC387904

[mmi14920-bib-0031] Iannelli, F. , Pearce, B.J. & Pozzi, G. (1999) The type 2 capsule locus of *Streptococcus pneumoniae* . Journal of Bacteriology, 181, 2652–2654. 10.1128/JB.181.8.2652-2654 10198036 PMC93698

[mmi14920-bib-0032] Iovino, F. , Nannapaneni, P. , Henriques‐Normark, B. & Normark, S. (2020) The impact of the ancillary pilus‐1 protein RrgA of *Streptococcus pneumoniae* on colonization and disease. Molecular Microbiology, 113, 650–658. 10.1111/mmi.14451 32185835

[mmi14920-bib-0033] Jensch, I. , Gamez, G. , Rothe, M. , Ebert, S. , Fulde, M. , Somplatzki, D. et al. (2010) PavB is a surface‐exposed adhesin of *Streptococcus pneumoniae* contributing to nasopharyngeal colonization and airways infections. Molecular Microbiology, 77, 22–43. 10.1111/j.1365-2958.2010.07189.x 20444103

[mmi14920-bib-0034] Kang, M.S. , Ko, Y.P. , Liang, X.W. , Ross, C.L. , Liu, Q. , Murray, B.E. et al. (2013) Collagen‐binding microbial surface components recognizing adhesive matrix molecule (MSCRAMM) of gram‐positive bacteria inhibit complement activation via the classical pathway. Journal of Biological Chemistry, 288, 20520–20531. 10.1074/jbc.M113.454462 23720782 PMC3711317

[mmi14920-bib-0035] Kanwal, S. , Jensch, I. , Palm, G.J. , Bronstrup, M. , Rohde, M. , Kohler, T.P. et al. (2017) Mapping the recognition domains of pneumococcal fibronectin‐binding proteins PavA and PavB demonstrates a common pattern of molecular interactions with fibronectin type III repeats. Molecular Microbiology, 105, 839–859. 10.1111/mmi.13740 28657670

[mmi14920-bib-0036] Kline, K.A. , Falker, S. , Dahlberg, S. , Normark, S. & Henriques‐Normark, B. (2009) Bacterial adhesins in host‐microbe interactions. Cell Host & Microbe, 5, 580–592. 10.1016/j.chom.2009.05.011 19527885

[mmi14920-bib-0037] Kostrzynska, M. & Wadstrom, T. (1992) Binding of laminin, type‐IV collagen, and vitronectin by *streptococcus‐pneumoniae* . Zentralblatt Fur Bakteriologie‐International Journal of Medical Microbiology Virology Parasitology and Infectious Diseases, 277, 80–83. 10.1016/s0934-8840(11)80874-1 1381646

[mmi14920-bib-0038] Kreikemeyer, B. , Nakata, M. , Oehmcke, S. , Gschwendtner, C. , Normann, J. & Podbielski, A. (2005) *Streptococcus pyogenes* collagen type I‐binding Cpa surface protein ‐ expression profile, binding characteristics, biological functions, and potential clinical impact. Journal of Biological Chemistry, 280, 33228–33239. 10.1074/jbc.M502896200 16040603

[mmi14920-bib-0039] Krishnan, V. & Narayana, S.V.L. (2011) Crystallography of gram‐positive bacterial adhesins. Bacterial Adhesion: Chemistry, Biology and Physics, 715, 175–195. 10.1007/978-94-007-0940-9_11 21557064

[mmi14920-bib-0040] Lentini, G. , Midiri, A. , Firon, A. , Galbo, R. , Mancuso, G. , Biondo, C. et al. (2018) The plasminogen binding protein PbsP is required for brain invasion by hypervirulent CC17 group B streptococci. Scientific Reports, 8, 14322. 10.1038/s41598-018-32774-8 30254272 PMC6156580

[mmi14920-bib-0041] Mackichan, J.K. , Gerns, H.L. , Chen, Y.T. , Zhang, P. & Koehler, J.E. (2008) A sacB mutagenesis strategy reveals that the *Bartonella quintana* variably expressed outer membrane proteins are required for bloodstream infection of the host. Infection and Immunity, 76, 788–795. 10.1128/IAI.01174-07 18070893 PMC2223462

[mmi14920-bib-0042] Merle, N.S. , Singh, P. , Rahman, J. & Kemper, C. (2021) Integrins meet complement: the evolutionary tip of an iceberg orchestrating metabolism and immunity. British Journal of Pharmacology, 178, 2754–2770. 10.1111/bph.15168 32562277 PMC8359198

[mmi14920-bib-0043] Nobbs, A.H. , Lamont, R.J. & Jenkinson, H.F. (2009) Streptococcus adherence and colonization. Microbiology and Molecular Biology Reviews, 73, 407–450. 10.1128/MMBR.00014-09 19721085 PMC2738137

[mmi14920-bib-0044] O'Brien, K.L. , Wolfson, L.J. , Watt, J.P. , Henkle, E. , Deloria‐Knoll, M. , Mccall, N. et al. (2009) Burden of disease caused by *Streptococcus pneumoniae* in children younger than 5 years: global estimates. Lancet, 374, 893–902. 10.1016/S0140-6736(09)61204-6 19748398

[mmi14920-bib-0045] Ogden, C.A. , DeCathelineau, A. , Hoffmann, P.R. , Bratton, D. , Ghebrehiwet, B. , Fadok, V.A. et al. (2001) C1q and mannose binding lectin engagement of cell surface calreticulin and CD91 initiates macropinocytosis and uptake of apoptotic cells. Journal of Experimental Medicine, 194, 781–795. 10.1084/jem.194.6.781 11560994 PMC2195958

[mmi14920-bib-0046] Oggioni, M.R. , Trappetti, C. , Kadioglu, A. , Cassone, M. , Iannelli, F. , Ricci, S. et al. (2006) Switch from planktonic to sessile life: a major event in pneumococcal pathogenesis. Molecular Microbiology, 61, 1196–1210. 10.1111/j.1365-2958.2006.05310.x 16925554 PMC1618759

[mmi14920-bib-0047] Papasergi, S. , Cariccio, V.L. , Pietrocola, G. , Domina, M. , D'aliberti, D. , Trunfio, M.G. et al. (2013) Immunogenic properties of Streptococcus agalactiae FbsA fragments. PLoS One, 8(9), e75266. 10.1371/journal.pone.0075266 24086487 PMC3782484

[mmi14920-bib-0048] Papasergi, S. , Garibaldi, M. , Tuscano, G. , Signorino, G. , Ricci, S. , Peppoloni, S. et al. (2010) Plasminogen‐ and fibronectin‐binding protein B is involved in the adherence of *Streptococcus pneumoniae* to human epithelial cells. Journal of Biological Chemistry, 285, 7517–7524. 10.1074/jbc.M109.062075 20048164 PMC2844199

[mmi14920-bib-0049] Patti, J.M. , Allen, B.L. , Mcgavin, M.J. & Hook, M. (1994) Mscramm‐mediated adherence of microorganisms to host tissues. Annual Review of Microbiology, 48, 585–617. 10.1146/annurev.mi.48.100194.003101 7826020

[mmi14920-bib-0050] Patti, J.M. & Hook, M. (1994) Microbial adhesins recognizing extracellular‐matrix macromolecules. Current Opinion in Cell Biology, 6, 752–758. 10.1016/0955-0674(94)90104-x 7833055

[mmi14920-bib-0051] Philominathan, S.T.L. , Koide, T. , Mathsushita, O. & Sakon, J. (2012) Bacterial collagen‐binding domain targets undertwisted regions of collagen. Protein Science, 21, 1554–1565. 10.1002/pro.2145 22898990 PMC3526996

[mmi14920-bib-0052] Picard, C. , Puel, A. , Bustamante, A. , Ku, C.L. & Casanova, J.L. (2003) Primary immunodeficiencies associated with pneumococcal disease. Current Opinion in Allergy and Clinical Immunology, 3, 451–459. 10.1097/00130832-200312000-00006 14612669

[mmi14920-bib-0053] Ricklin, D. , Hajishengallis, G. , Yang, K. & Lambris, J.D. (2010) Complement: a key system for immune surveillance and homeostasis. Nature Immunology, 11, 785–797. 10.1038/ni.1923 20720586 PMC2924908

[mmi14920-bib-0054] Sarma, V.J. , Huber‐Lang, M. & Ward, P.A. (2006) Complement in lung disease. Autoimmunity, 39, 387–394. 10.1080/08916930600739456 16923538

[mmi14920-bib-0055] Shoemaker, N.B. & Guild, W.R. (1974) Destruction of low efficiency markers is a slow process occurring at a heteroduplex stage of transformation. Molecular Genetics and Genomics, 128, 283–290. 10.1007/BF00268516 4150368

[mmi14920-bib-0056] Sillanpaa, J. , Nallapareddy, S.R. , Qin, X. , Singh, K.V. , Muzny, D.M. , Kovar, C.L. et al. (2009) A collagen‐binding adhesin, Acb, and ten other putative MSCRAMM and pilus family proteins of *streptococcus gallolyticus* subsp gallolyticus (*Streptococcus bovis* group, biotype I). Journal of Bacteriology, 191, 6643–6653. 10.1128/jb.00909-09 19717590 PMC2795296

[mmi14920-bib-0057] Singh, B. , Fleury, C. , Jalalvand, F. & Riesbeck, K. (2012) Human pathogens utilize host extracellular matrix proteins laminin and collagen for adhesion and invasion of the host. Fems Microbiology Reviews, 36, 1122–1180. 10.1111/j.1574-6976.2012.00340.x 22537156

[mmi14920-bib-0058] Speziale, P. , Arciola, C.R. & Pietrocola, G. (2019) Fibronectin and its role in human infective diseases. Cells, 8(12), 1516. 10.3390/cells8121516 PMC6952806 31779172

[mmi14920-bib-0059] Strawich, E. & Nimni, M.E. (1971) Properties of a collagen molecule containing three identical components extracted from bovine articular cartilage. Biochemistry, 10(21), 3905–3911. 10.1021/bi00797a017 4334282

[mmi14920-bib-0060] Terrasse, R. , Amoroso, A. , Vernet, T. & Di Guilmi, A.M. (2015) Streptococcus pneumoniae GAPDH is released by cell lysis and interacts with peptidoglycan. PLoS One, 10(4), e0125377. 10.1371/journal.pone.0125377 25927608 PMC4415926

[mmi14920-bib-0061] Terrasse, R. , Tacnet‐Delorme, P. , Moriscot, C. , Perard, J. , Schoehn, G. , Vernet, T. et al. (2012) Human and pneumococcal cell surface glyceraldehyde‐3‐phosphate dehydrogenase (GAPDH) proteins are both ligands of human C1q protein. Journal of Biological Chemistry, 287, 42620–42633. 10.1074/jbc.M112.423731 23086952 PMC3522263

[mmi14920-bib-0062] Valotteau, C. , Prystopiuk, V. , Pietrocola, G. , Rindi, S. , Peterle, D. , De Filippis, V. et al. (2017) Single‐cell and single‐molecule analysis unravels the multifunctionality of the *Staphylococcus aureus* collagen‐binding protein Cna. ACS Nano, 11, 2160–2170. 10.1021/acsnano.6b08404 28151647

[mmi14920-bib-0063] Vassal‐Stermann, E. , Lacroix, M. , Gout, E. , Laffly, E. , Pedersen, C.M. , Martin, L. et al. (2014) Human L‐ficolin recognizes phosphocholine moieties of pneumococcal teichoic acid. Journal of Immunology, 193, 5699–5708. 10.4049/jimmunol.1400127 25344472

[mmi14920-bib-0064] Wagner, C. , Khan, A.S. , Kamphausen, T. , Schmausser, B. , Unal, C. , Lorenz, U. et al. (2007) Collagen binding protein Mip enables *legionella pneumophila* to transmigrate through a barrier of NCI‐H292 lung epithelial cells and extracellular matrix. Cellular Microbiology, 9, 450–462. 10.1111/j.1462-5822.2006.00802.x 16953800

[mmi14920-bib-0065] Xu, Y. , Liang, X.W. , Chen, Y.H. , Koehler, T.M. & Hook, M. (2004) Identification and biochemical characterization of two novel collagen binding MSCRAMMs of *bacillus anthracis* . Journal of Biological Chemistry, 279, 51760–51768. 10.1074/jbc.M406417200 15456768

[mmi14920-bib-0066] Xue, Q.O. , Gu, C.F. , Rivera, J. , Hook, M. , Chen, X.W. , Pozzi, A. et al. (2011) Entry of *bacillus anthracis* spores into epithelial cells is mediated by the spore surface protein BclA, integrin alpha 2 beta 1 and complement component C1q. Cellular Microbiology, 13, 620–634. 10.1111/j.1462-5822.2010.01558.x 21134100

