# Supplementary figures and images for: Streptococcus pneumoniae binds collagens and C1q via the SSURE repeats of the PfbB adhesin
Source: Mol Microbiol. 2022 May 30;117(6):1479–92. doi: 10.1111/mmi.14920 (PMC9328315; doi:10.1111/mmi.14920)

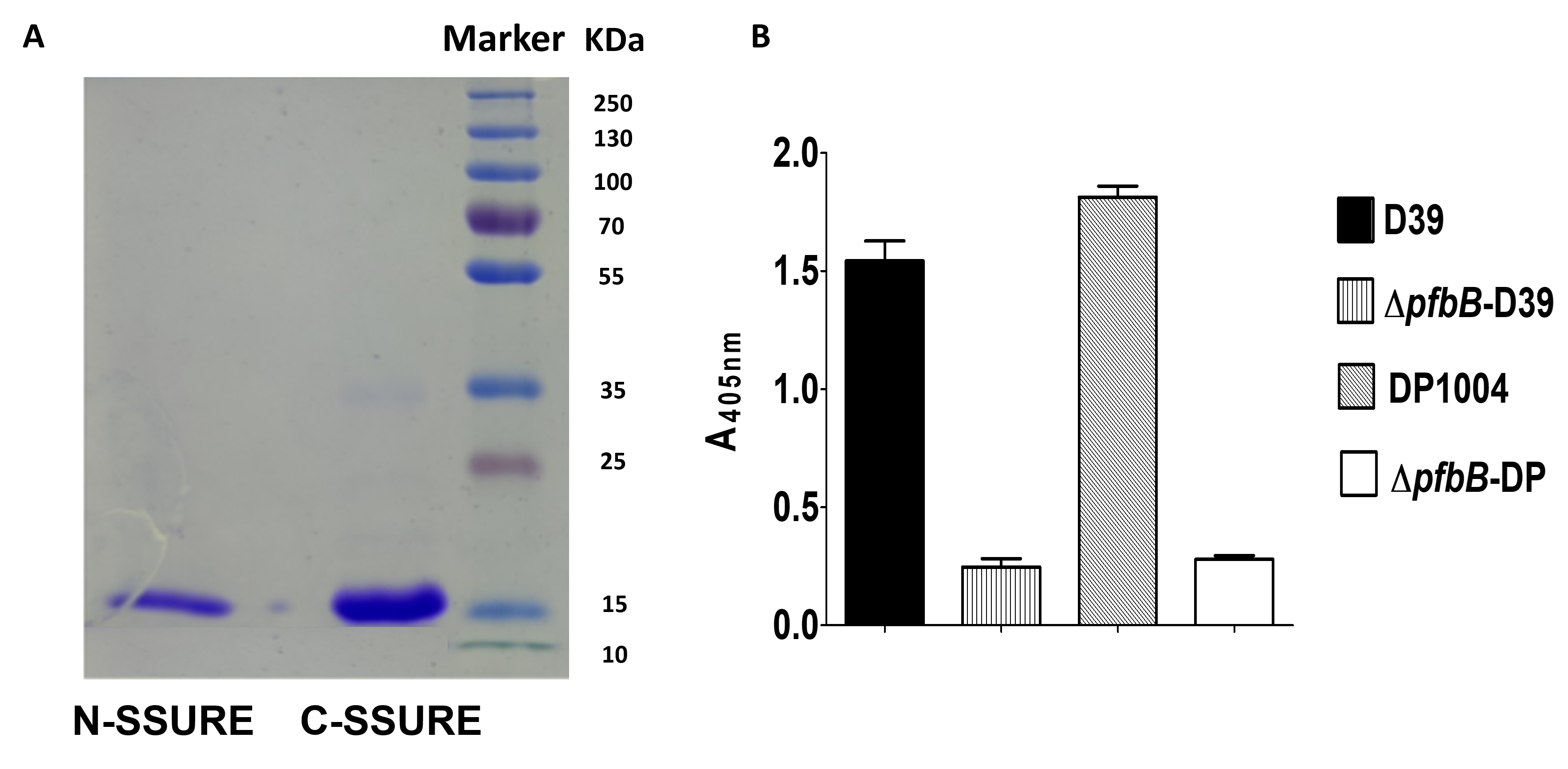

Supplement: Supplementary file 2 — Figure S2 [file MMI-117-1479-s002.tif]
