# Supplementary material for: Streptococcus pneumoniae binds collagens and C1q via the SSURE repeats of the PfbB adhesin
Source: Mol Microbiol. 2022 May 30;117(6):1479–92. doi: 10.1111/mmi.14920 (PMC9328315; doi:10.1111/mmi.14920)
Supplement: Supplementary file 3 — FigureCaption [file MMI-117-1479-s003.docx]

**Figure** **S1** **Unencapsulated *S. pneumoniae* grows in sessile form on surfaces coated with type I collagen by a mechanism involving PfbB**. Effect of *pfbB* deletion on sessile growth of the unencapsulated DP1004 strain in wells coated with type I collagen (Coll I) or bovine serum albumin (BSA). Δ*pfbB*-DP, *pfbB* deletion mutant of the DP1004 strain. Columns indicate optical density at 595 nm (OD_595nm_) of solubilized crystal violet. Shown are means ± SDs of three independent experiments conducted in triplicate. *, p < 0.05 by the Mann–Whitney test.

**Figure** **S2 Purity of recombinant SSURE fragments and PfbB-specificity of the anti-SSURE serum. (A)** Coomassie staining of purified recombinant histidine-tagged N- and C-SSURE fragments, separated by polyacrylamide gel electrophoresis. (**B**) PfbB-specificity of anti-SSURE serum assessed by measuring reactivity with wild type and *pfbB*-deleted encapsulated and unencapsulated pneumococci. Binding of anti-SSURE antibodies on wells coated with bacteria was revealed by an ELISA assay. Δ*pfbB*-D39, *pfbB* deletion mutant of D39 encapsulated pneumococci. Δ*pfbB*-DP, *pfbB* deletion mutant of DP1004 unencapsulated pneumococci. Shown are means ± SDs of three independent experiments conducted in triplicate.
